# Nuclear reorganization by NPM1-mediated phase separation triggered by adenovirus core protein VII

**DOI:** 10.1128/spectrum.00416-24

**Published:** 2024-08-20

**Authors:** Michelle Jane Genoveso, Mitsuru Okuwaki, Kohsuke Kato, Kyosuke Nagata, Atsushi Kawaguchi

**Affiliations:** 1 Department of Infection Biology, Institute of Medicine, University of Tsukuba, Tsukuba, Japan; 2 Laboratory of Biochemistry, School of Pharmacy, Kitasato University, Tokyo, Japan; 3 Transborder Medical Research Center, University of Tsukuba, Tsukuba, Japan; 4 Microbiology Research Center for Sustainability, University of Tsukuba, Tsukuba, Japan; 5 Center for Quantum and Information Life Sciences, University of Tsukuba, Tsukuba, Japan; Oklahoma State University College of Veterinary Medicine, Stillwater, Oklahoma, USA

**Keywords:** adenovirus, LLPS, NPM1, nucleolus, ViPR body

## Abstract

Recent evidence has revealed that the reorganization of nuclear domains is largely mediated by liquid-liquid phase separation (LLPS). During viral infection, numerous nuclear domains undergo significant changes through LLPS for and against the replication of the virus. However, the regulatory mechanism of LLPS in response to viral infection and its detailed functions in viral replication remain unclear. In this study, we found that the activity of the nucleolar protein NPM1, a remodeling factor for the chromatin-like structure of adenovirus DNA, to induce LLPS is required for deposition of adenovirus core protein VII in a subnuclear domain, the virus-induced post-replication (ViPR) body, in the late phases of infection. The interaction between NPM1 and protein VII was responsible for initiating LLPS. The inhibition of LLPS by 1,6-hexanediol treatment resulted in the dispersion of protein VII from the ViPR bodies. These findings suggest that protein VII accumulates in the ViPR bodies in concert with the LLPS formation of NPM1 triggered by protein VII. After photobleaching of EGFP-NPM1 in the ViPR bodies, EGFP-NPM1 showed a relatively fast recovery half-time, indicating the fluid-like properties of NPM1 in this compartment. Importantly, NPM1 depletion decreased the genome packaging in the viral capsids, possibly owing to the formation of a defective adenovirus core. This study highlights the dynamic interplay between viral pathogens and the host nucleus for the reorganization of membrane-less compartments that facilitate their replication.

**IMPORTANCE:**

In this study, we explored how adenoviruses utilize a process known as liquid-liquid phase separation (LLPS) to enhance their replication. We focused on a cellular chromatin remodeling protein, NPM1, which plays a crucial role in nucleolar formation through LLPS. NPM1 facilitates LLPS by interacting with adenovirus protein VII, effectively accumulating protein VII into membrane-less compartments called virus-induced post-replication bodies. NPM1 functions as a molecular chaperone of protein VII to assemble viral chromatin by transferring protein VII to viral DNA. Remarkably, when NPM1 was depleted, this process was disrupted, decreasing viral genome packaging. These findings shed light on a critical aspect of virus-host interactions, illustrating how adenovirus utilizes NPM1-mediated LLPS activity. Our findings provide valuable insights into the dynamic interplay between viruses and the host nucleus.

## INTRODUCTION

The nucleus is highly compartmentalized into numerous distinct nuclear compartments such as the nucleolus and Cajal bodies. Recent reports show that most nuclear compartments are not separated by a cellular membrane and are organized by a spontaneous process known as liquid-liquid phase separation (LLPS) (1, 2). In LLPS, a homogeneous solution spontaneously separates into two distinct states with different solute concentrations (3, 4). LLPS is a dynamic and reversible process that is pivotal in the formation of biomolecular condensates and is implicated not only in compartmentalization of nucleic acids/proteins (5) but also in adaptive responses (6) and transcriptional control (7).

One of the well-studied LLPS-formed nuclear organelles is the nucleolus (8–11), a vital center for ribosomal RNA (rRNA) synthesis and ribosomal subunit assembly. Nucleolar activity is controlled in response to several cellular responses, such as apoptosis, DNA damage response, cellular proliferation, and cellular senescence (12, 13). Notably, key proteins, including nucleophosmin 1 (NPM1) and fibrillarin (FBL), have been shown to undergo LLPS and accumulate within the nucleolus and to function actively in ribosomal RNA transcription and processing and in ribosome assembly (8, 14–16). Previous reports have demonstrated that viral infections induce substantial changes to the nucleolus, including targeted recruitment of viral proteins, redistribution of resident nucleolar proteins to viral replication machineries, and translocation of non-nucleolar host proteins into the nucleolus (16–21). This reorganization involves various nucleolar proteins such as NPM1, UBF, fibrillarin, nucleolin, POLD1, TCOFI, NOLC1, and SUMO1 (22–24). For instance, Adenovirus infection has been shown to induce significant alterations to the nucleolus (25–30). The adenovirus (Ad) genome is a linear, double-stranded DNA associated with viral basic proteins, such as proteins V, VII, and μ (31–34). Protein VII is a major core protein and assembles Ad DNA into a nucleosome-like structure called the Ad core (35–37). In the early phases of infection, replication of Ad DNA takes place within nuclear foci containing DBP, a viral single-stranded DNA-binding protein. During these early phases, the replicated Ad DNA associates with not only core proteins but also cellular histones for viral gene expression (38). The replicated Ad DNA then accumulates in virus-induced post-replication (ViPR) bodies, from which the cellular histones are removed by an unknown mechanism (39). Although the exact function of the ViPR bodies is unclear, protein VII and cellular chromatin remodeling proteins, including NPM1, reportedly colocalize in the ViPR bodies, suggesting that they may serve as a nuclear domain crucial for viral genome remodeling before packaging (23). Notably, NPM1 is required to form ViPR bodies (23) and functions as a molecular chaperone of protein VII (40). It is also reported that in the late phases of infection, the Ad core is packaged into the capsids, which are densely arranged in the nucleus in a honeycomb-like organization called the late virion accumulation compartment (LVAC) (41).

Emerging lines of evidence highlight how viruses exploit LLPS to their advantage in promoting their replication (42–44). During adenovirus infection, phase-separated nuclear compartments are formed to serve as reservoir for viral genome replication (45), to facilitate production of infectious viral particles (46) and to assure efficiency of viral genome packaging (47). In this study, we found that protein VII is deposited in ViPR bodies through LLPS mediated by the nucleolar protein NPM1. Our analyses also revealed that the interaction between NPM1 and protein VII triggers heterotypic LLPS *in vitro*. Notably, NPM1 depletion decreased genome packaging into the viral capsids and disrupted the formation of the LVAC. The mechanistic dissection of these dynamic events has shed light on the interplay between viral pathogens and the host nucleus, especially furthering the understanding of how adenovirus hijacks the LLPS activity of NPM1 to organize viral replication.

## RESULTS

### The LLPS protein, NPM1, accumulates in ViPR bodies

**Fig 1:**
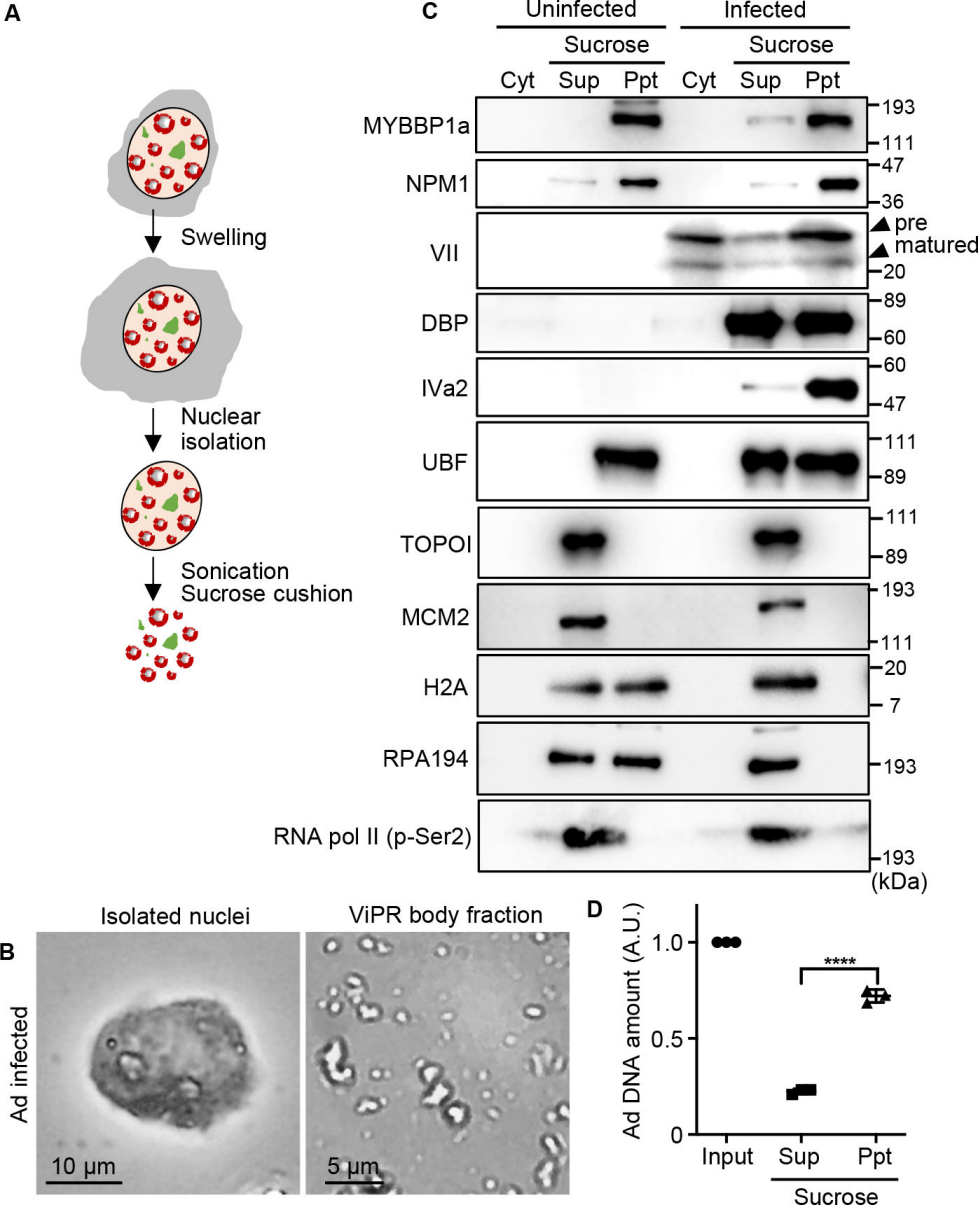
Ad ViPR body isolation. (**A**) Overview of ViPR body isolation. Swollen Ad-infected U2OS cells were homogenized and centrifuged to isolate the nuclei at 36 h post-infection. The isolated nuclei were sonicated and fractionated through a sucrose cushion centrifugation. (**B**) Brightfield microscopy images of isolated nuclei and partially isolated ViPR body fraction. Scales are indicated in each picture. (**C**) Western blot analysis of the cytoplasmic (Cyt), supernatant (Sup), and precipitate (Ppt) fractions with indicated antibodies. (**D**) Quantification of Ad DNA amounts using quantitative PCR (qPCR) assay. The amounts of Ad DNA in the input, supernatant (Sup), and precipitate (Ppt) fractions isolated at 36 h post-infection were quantified. Viral DNA was amplified by qPCR using primers specific for Ad *E1A* gene region. The amount of viral DNA in the input was set to 1.0. The data show three independent experiments performed in duplicates. Error bars indicate ±SD. *P* values were calculated using Student’s *t*-test (****P < 0.0001).

In the late phases of infection (24 to 36 h post-infection), ViPR bodies are observed as large nuclear foci containing DNA and surrounded by DBP-positive ring-like structures (39). Because ViPR bodies contain low amounts of cellular histones and high amounts of protein VII, the Ad core likely accumulates in ViPR bodies after viral DNA replication (38). To elucidate the function of ViPR bodies in the Ad life cycle, we conducted proteomics analysis using purified ViPR body-containing fractions (Fig. 1A). Ad-infected U2OS cells were collected at 36 h post-infection, allowed to swell in hypotonic buffer, and homogenized by use of a Dounce homogenizer. After centrifugation, the isolated nuclei were sonicated and subjected to two-step sucrose cushion centrifugation. Brightfield observation showed that organelle-like structures were purified from the pellet fraction of the sucrose centrifugation (Fig. 1B). Although DBP was detected in both the supernatant and the pellet fractions of the sucrose centrifugation, proteins VII and IVa2 were observed in the pellet fraction together with host nucleolar proteins, including UBF and NPM1, previously reported to localize in ViPR bodies (Fig. 1C) (23, 39). In addition, a ViPR body molecular marker, Mybbp1a (48), was also detected in the pellet fractions from Ad-infected cells, suggesting it mainly contains ViPR bodies. It is reported that although ViPR bodies contain highly dense DNA, histone H2A does not accumulate in them (40). Consistent with this finding, histone H2A was detected in the pellet fraction obtained from the uninfected cells, but not in that from the Ad-infected cells (Fig. 1C). The purification method used in this study is similar to the isolation protocols used for the nucleolus and Ad replication compartments (25, 49–51). Thus, it was assumed that some of the nucleolar proteins and host factors enriched in Ad replication compartments were cofractionated with the ViPR bodies. However, RPA194 was not detected, suggesting that few nucleolar proteins were cofractionated in the pellet fraction. This is possibly due to the destruction of the nucleolus at 36 h post-infection. Furthermore, host factors previously reported to be enriched in isolated Ad replication compartments, such as MCM2, TOPOI, and RNA pol II CTD Ser2 phosphorylation (52), were not enriched in the ViPR body-containing fraction, suggesting that replication compartments were hardly co-fractionated with the ViPR bodies. The amount of viral DNA was also enriched in the pellet fraction (Fig. 1D).

**Fig 2:**
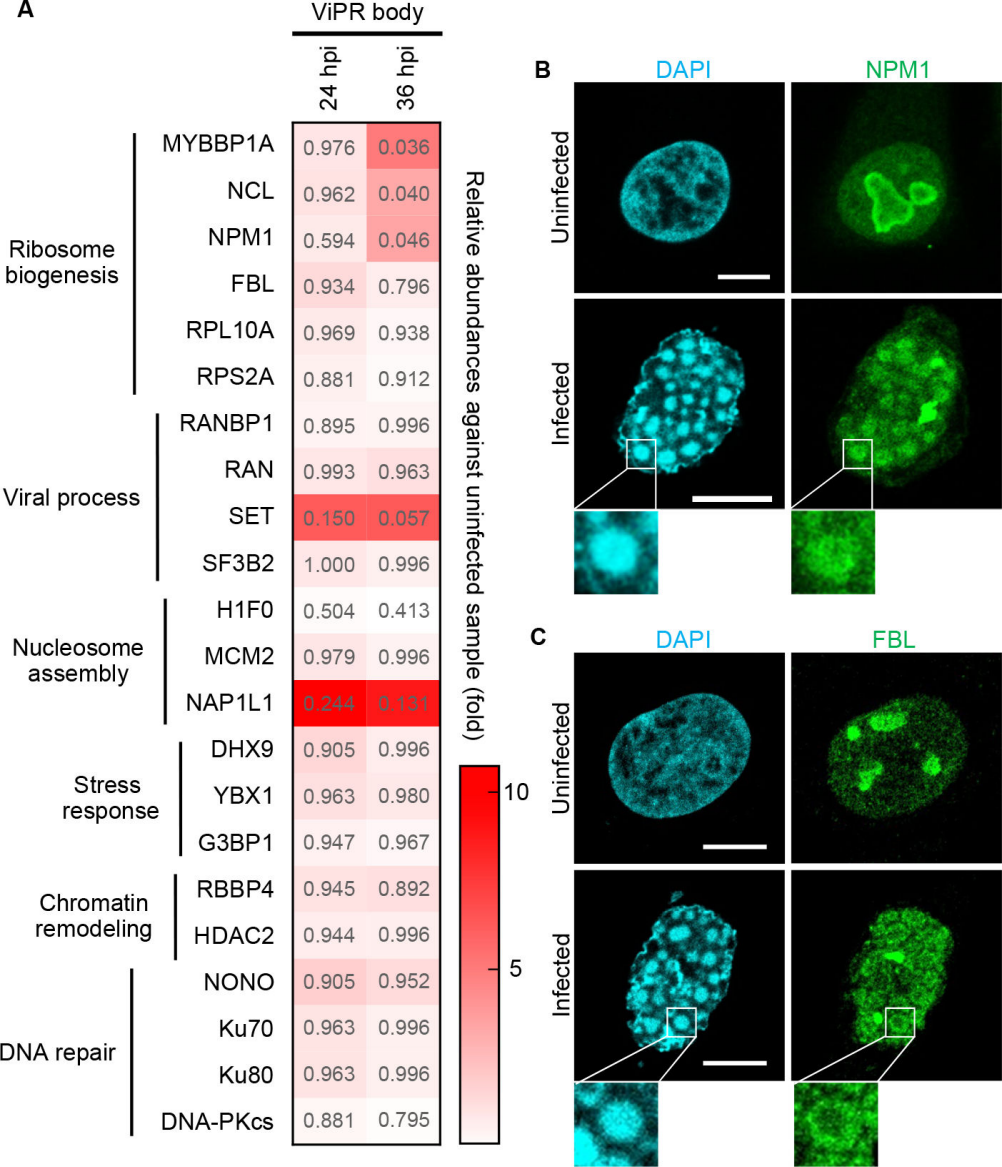
The LLPS protein, NPM1, accumulates in the ViPR bodies. Gene Ontology annotations of partially isolated ViPR body fraction at 24 and 36 h post-infection carried out using the DAVID database. (**A**) The nuclear proteins that were consistently detected in each of the measurements were categorized by Gene Ontology annotations using the DAVID database. The relative peptide abundance obtained at 24 or 36 hpi against that of uninfected sample was shown as a heatmap. Each *P* value was shown in the heatmap. Uninfected and Ad-infected U2OS cells were subjected to indirect immunofluorescence assays at 36 h post-infection with anti-NPM1 (**B**) and anti-FBL (**C**) antibodies, respectively. DAPI was used to stain DNA. Scale bars indicate 10 µm. Enlarged images showing the accumulation of each protein in the ViPR bodies are shown at the bottom panel.

The isolated fraction containing ViPR bodies was analyzed by liquid chromatography-mass spectrometry to identify the proteins contained. In this experiment, ViPR body-containing fractions were isolated from Ad-infected U2OS cells at 24 and 36 h post-infection. The nuclear proteins that were consistently detected were categorized with Gene Ontology (GO) annotations by DAVID (Database for Annotation, Visualization and Integrated Discovery). The relative abundances of peptides detected at 24 or 36 h post-infection to these in the uninfected samples were plotted as a heatmap (Fig. 2A). The GO analysis showed that proteins detected in the ViPR body-containing fractions were involved in ribosome biogenesis, viral replication, DNA replication, intracellular viral transport, nucleosome assembly, protein localization to cytoplasmic stress granules, chromatin remodeling, and DNA repair. However, consistent with Fig. 1B, Mybbp1a, is enriched in the ViPR body-containing fractions (Fig. 2A). The amounts of nucleolar proteins such as NPM1 and NCL were also significantly enriched.

Among these proteins, we focused on the nucleolar proteins NPM1 and FBL due to their LLPS activities. NPM1 induces both homotypic and heterotypic LLPS through its multivalent interactions (53, 54). NPM1 is also reported to bind to arginine-rich proteins via its negatively charged acidic tracts, forming heterotypic liquid droplets (55). FBL contains a glycine-arginine-rich domain (GAR-domain) in its N-terminal intrinsically disordered region, which directs the protein to the nucleolus. The dysregulation of FBL could impact the LLPS maintenance of the nucleolar structure (15). To examine the accumulation of NPM1 and FBL in ViPR bodies, we performed indirect immunofluorescence assays using anti-NPM1 (Fig. 2B) and anti-FBL (Fig. 2C) antibodies, respectively. In the uninfected cells, NPM1 was mostly localized in the nucleolar periphery (Fig. 2B), while FBL was in the center (Fig. 2C). In Ad-infected cells, NPM1 colocalized well with the highly dense DNA foci in the center of the ViPR bodies as previously reported (23) (Fig. 2B), while FBL formed irregular ring-like structures outside the ViPR bodies and did not colocalize with the DNA foci (Fig. 2C). This suggests that the nucleolar localization of NPM1 and FBL is reversed in that of the ViPR body.

### Accumulation of NPM1 in ViPR bodies is mediated by LLPS

**Fig 3:**
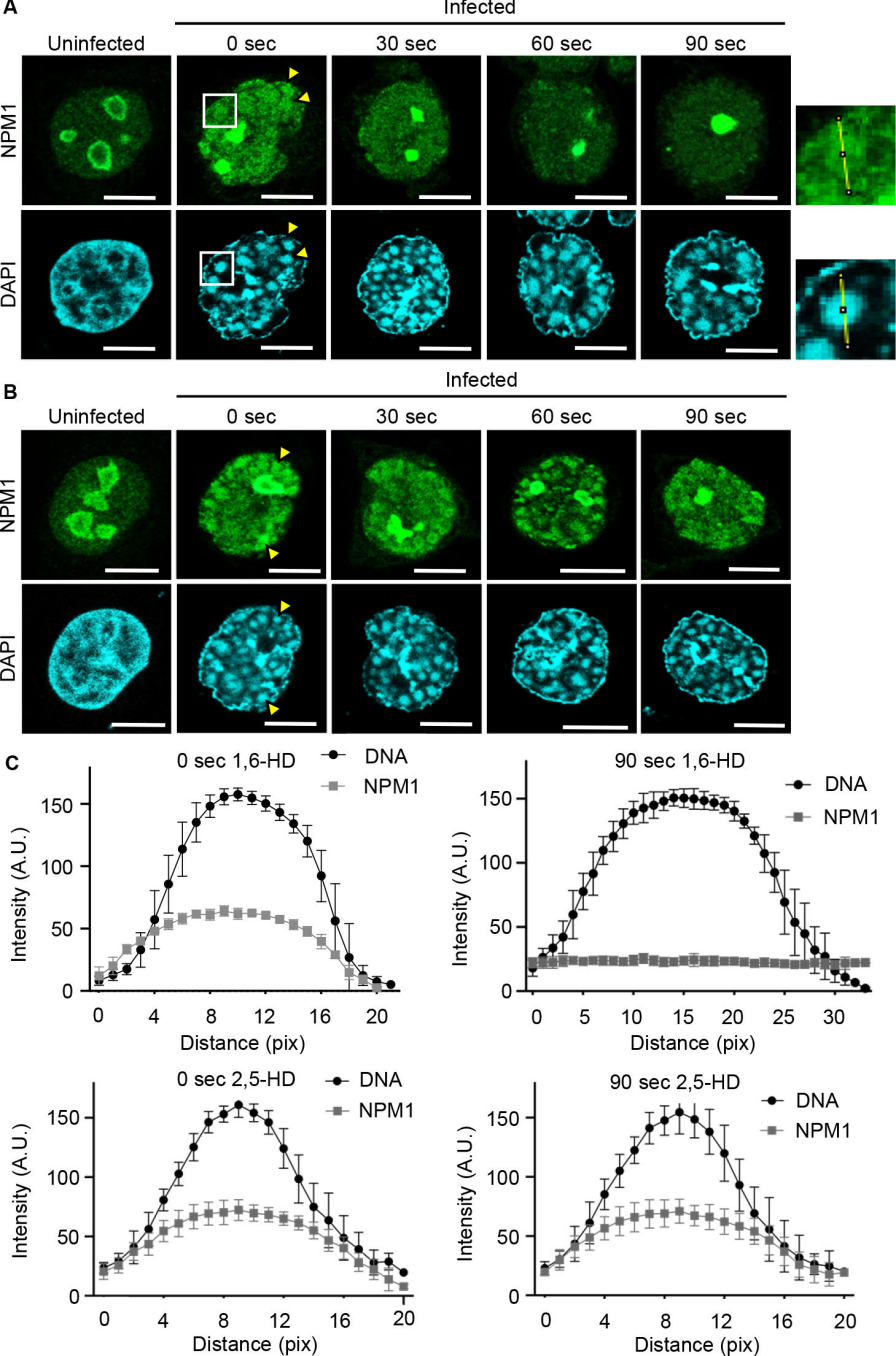
The accumulation of NPM1 in the ViPR bodies by LLPS. At 36 h post-infection, uninfected and Ad-infected U2OS cells were subjected to indirect immunofluorescence assays followed by either 3.8% 1,6-HD (**A**) or 2,5-HD (**B**) treatment for 0, 30, 60, and 90 sec. The cells were stained with DAPI and anti-NPM1 antibody. Scale bars, 10 µm. Yellow arrowheads indicate NPM1 and DNA accumulation foci in the ViPR bodies. (**C**) Fluorescence intensity line profile analysis of ViPR bodies treated with either 3.8% 1,6-HD (upper panels) or 2,5-HD (lower panels). Each graph shows DNA and NPM1 fluorescence intensity line profiles. The start point (0 pix) on the profile graph corresponds to the start point of the line drawn across a ViPR body as shown at the upper immunostaining images. Ten cells per condition were analyzed. From each cell, three ViPR bodies were measured and plotted. Standard deviations of 30 ViPR bodies are shown.

To test whether LLPS mediates the accumulation of NPM1 in ViPR bodies, we next treated Ad-infected cells with either 1,6-hexanediol (1,6-HD) or 2,5-hexanediol (2,5-HD). Previous studies showed that 2%–10% 1,6-HD disrupts phase-separated nuclear condensates by interfering with weak hydrophobic interactions; by contrast, 2,5-HD, a less hydrophobic derivative of 1,6-hexanediol, hardly dissolves liquid droplets, even at high concentrations (56). To avoid off-target responses, we used low concentrations of 1,6-HD (3.8%) along with short treatment time (maximum 90 sec) to enable examination of specific effects of the conditions applied. As indicated in Fig. 2B, NPM1 was initially localized in the ViPR bodies in the absence of 3.8% 1,6-HD (Fig. 3A and B; arrowheads). Interestingly, NPM1 was dispersed from the ViPR bodies to the nucleoplasm in a time-dependent manner by 1,6-HD treatment (Fig. 3A), but not by 2,5-HD treatment (Fig. 3B). To obtain quantitative data, we analyzed the line-scanning profiles of fluorescence intensity for DNA foci (ViPR bodies) and NPM1 (Fig. 3C). The fluorescence intensity profile of NPM1 exhibited a distinct peak at 0 sec after 1,6-HD treatment, which gradually flattened with prolonged exposure (Fig. 3C, upper panels); by contrast, no changes were observed in the fluorescence intensity profile of NPM1 in the presence of 2,5-HD treatments (Fig. 3C, lower panels).

**Fig 4:**
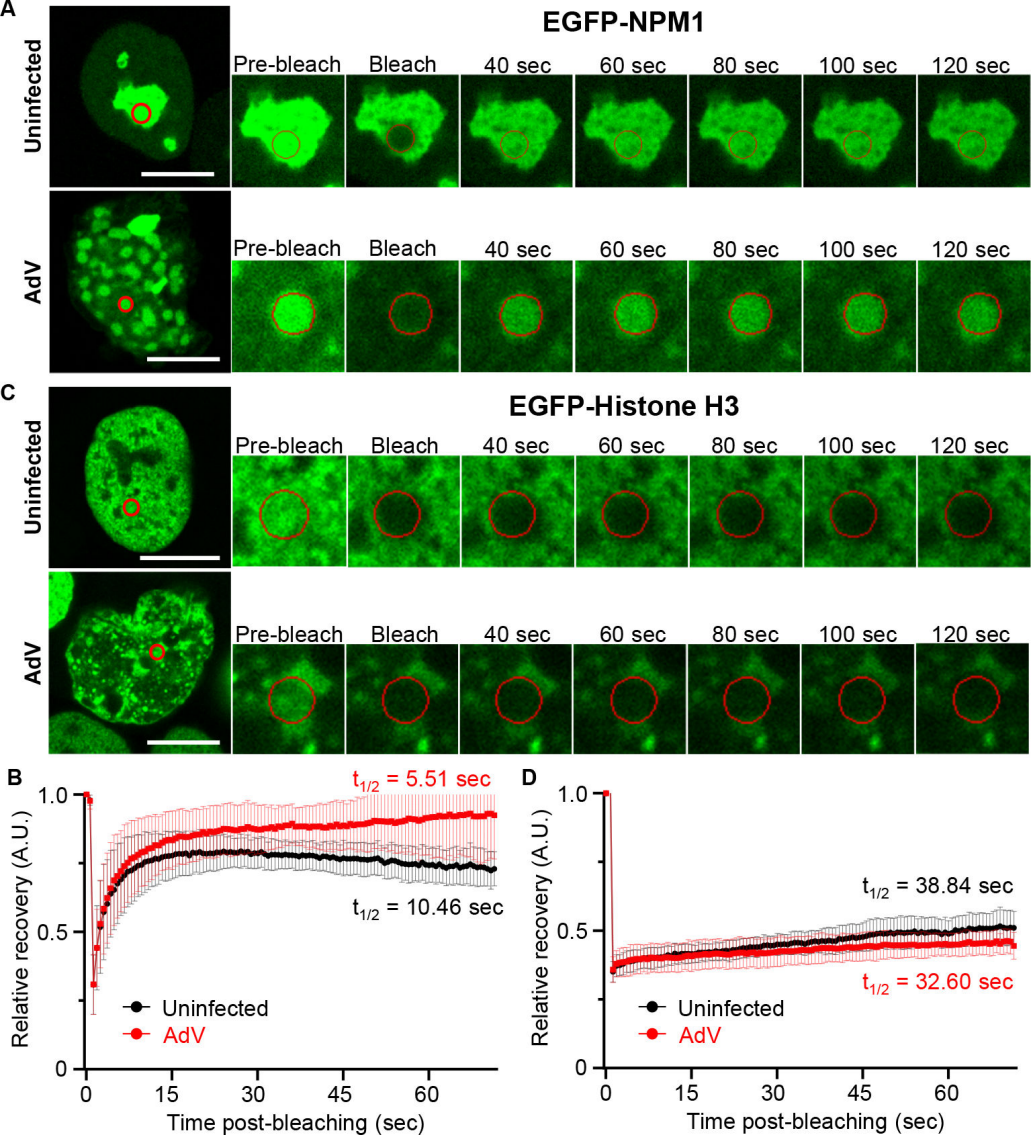
EGFP-NPM1 is highly mobile in the ViPR bodies. HeLa cells expressing EGFP-NPM1 or EGFP-H3 were infected with AdV and subjected to FRAP assays. (**A and C**) Representative images before, during, and after photobleaching in a 20-sec interval are shown. (**B and D**) Relative fluorescence intensities of the bleached regions against the non-bleached regions are shown. The mean half-recovery time was calculated using GraphPad Prism software. For each sample, 10 cells were photobleached and the data were averaged. Error bars indicate ±SD. Scale bar indicates 10 µm.

To strengthen the notion that the accumulation of NPM1 in the ViPR bodies is mediated by LLPS, we carried out fluorescence recovery after photobleaching (FRAP) assays on Ad-infected cells. LLPS is characterized by the formation of droplets with biomolecular components that typically exhibit rapid fluorescence recovery in FRAP assays (57). HeLa cells stably expressing EGFP-NPM1 were infected with AdV and subjected to FRAP assays at 36 h post-infection. In uninfected cells, EGFP-NPM1 in the nucleolus showed a relatively fast fluorescence recovery half-time of 10.46 sec (Fig. 4A and B). As expected, EGFP-NPM1 in the ViPR bodies also exhibited a rapid recovery half-time of 5.51 sec (Fig. 4A and B). As a control, we also performed FRAP assays with HeLa cells constitutively expressing EGFP-Histone H3, which stably binds to chromatin and is excluded from the ViPR bodies (Fig. 4C and D). The fluorescence recovery rate of EGFP-Histone H3 was observed to be very slow and comparable in uninfected and infected cells with recovery half-times of 38.84 and 32.60 sec, respectively (Fig. 4D). These results suggest that although NPM1 accumulates in the ViPR bodies, it remains highly mobile and may undergo LLPS, similar to NPM1 in the nucleolus.

### Localization of protein VII, but not of IVa2, in ViPR bodies is mediated by LLPS

**Fig 5:**
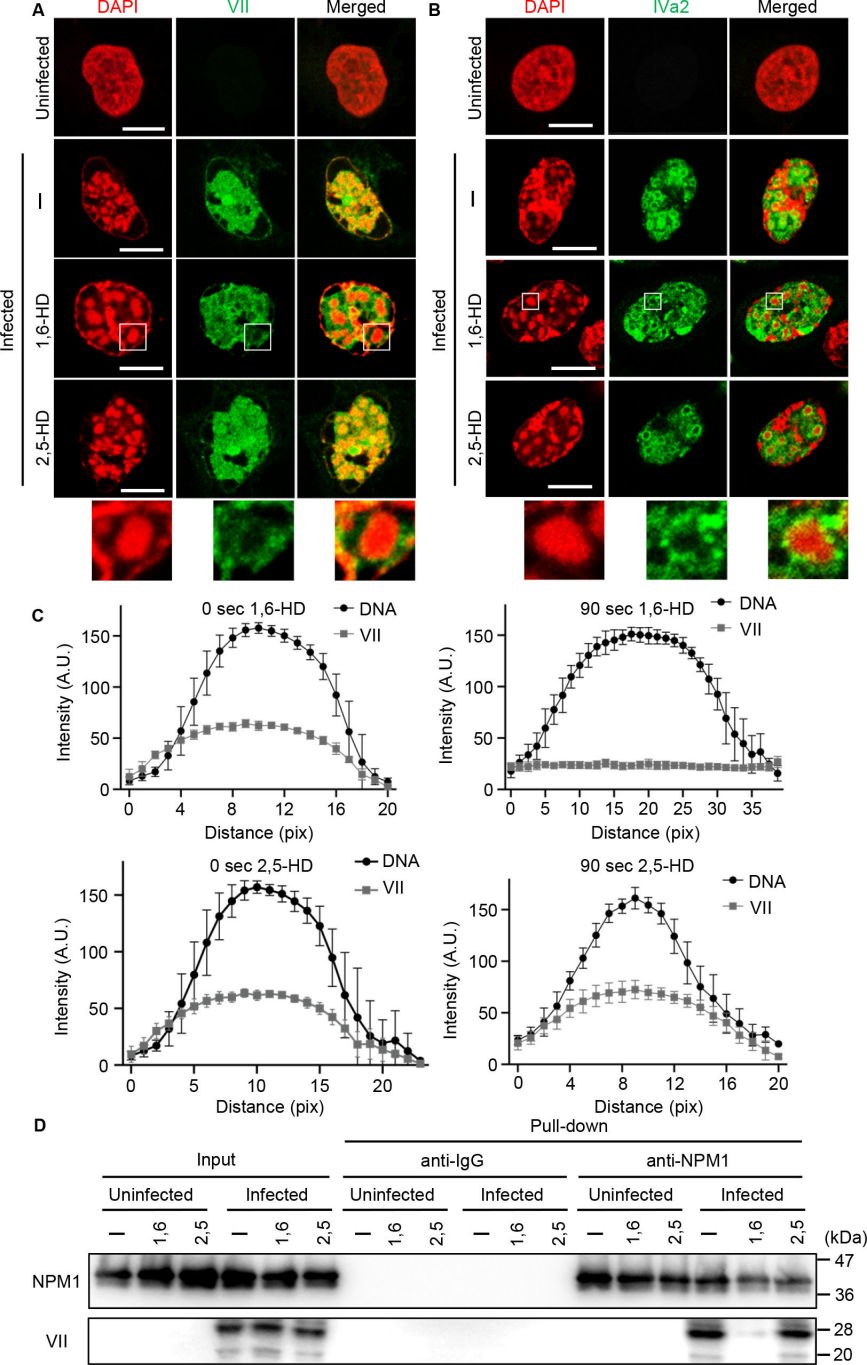
Protein VII, but not IVa2, localizes in the ViPR bodies by LLPS. (**A and B**) At 36 h post-infection, uninfected and Ad-infected U2OS cells were incubated with either 3.8% 1,6-HD or 2,5-HD for 90 sec. Indirect IF analysis with anti-VII (**A**) and anti-IVa2 (**B**) antibodies was carried out after HD treatments. DAPI staining shows DNA localization in red. Merged images are shown in the right panel. Scale bars, 10 µm. Enlarged images showing the localization of each protein in the ViPR body after 1,6-HD treatment are shown. (**C**) Fluorescence intensity line profile analysis of ViPR bodies treated with either 3.8% 1,6-HD (upper panels) or 2,5-HD (lower panels). Each graph shows DNA and protein VII fluorescence intensity line profiles. The start point (0 pix) on the profile graph corresponds to the start point of the line drawn across a ViPR body as shown at the upper immunostaining images. Ten cells per condition were analyzed. From each cell, three ViPR bodies were measured and plotted. Standard deviations of 30 ViPR bodies are shown. (**D**) Uninfected and Ad-infected cell lysates were immunoprecipitated with anti-IgG and anti-NPM1 antibodies, respectively. Immunoprecipitated proteins were subjected to western blot analysis with anti-NPM1 and anti-VII antibodies, respectively.

The replicated Ad DNA genome is translocated from the replication foci to the ViPR bodies and colocalized with viral core proteins, such as proteins VII, V, and IVa2 (23, 31, 39). To determine whether these viral proteins are deposited in the ViPR bodies by LLPS, we examined the intracellular localization of protein VII and IVa2 upon 1,6-HD treatment. As previously reported, protein VII accumulated with the ViPR bodies at 36 h post-infection (Fig. 5A). Interestingly, 1,6-HD treatment resulted in the relocalization of protein VII from the ViPR bodies to the nucleoplasm (Fig. 5A, enlarged panel). In contrast, protein IVa2 formed a ring-like staining pattern at the periphery of the ViPR bodies (Fig. 5B). Neither 3.8% 1,6-HD nor 2,5-HD treatment altered the localization pattern of protein IVa2. These results suggest that protein VII, but not protein IVa2, is deposited in the ViPR bodies by LLPS.

To obtain quantitative data, we also analyzed the line-scanning profiles of fluorescence intensity for DNA foci (ViPR bodies) and protein VII (Fig. 5C). The fluorescence intensity profile of protein VII exhibited a distinct peak at 0 sec after 1,6-HD treatment, which gradually flattened with prolonged exposure (Fig. 5C, upper panels); in contrast, no changes were observed in the fluorescence intensity profile of protein VII in the presence of 2,5-HD treatments (Fig. 5C, lower panels).

To further examine the LLPS-mediated accumulation of protein VII with NPM1 in Ad-infected cells, we next performed immunoprecipitation assays with anti-NPM1 antibodies with either 3.8% 1,6-HD or 2,5-HD treatment (Fig. 5D). The amount of protein VII was reduced by 1,6-HD treatment when compared with that of the control cells; however, this was not the case with 2,5-HD treatment. These results indicate that the LLPS of NPM1 is crucial for the deposition of protein VII in ViPR bodies.

### Protein VII triggers heterotypic LLPS of NPM1 *in vitro*

**Fig 6:**
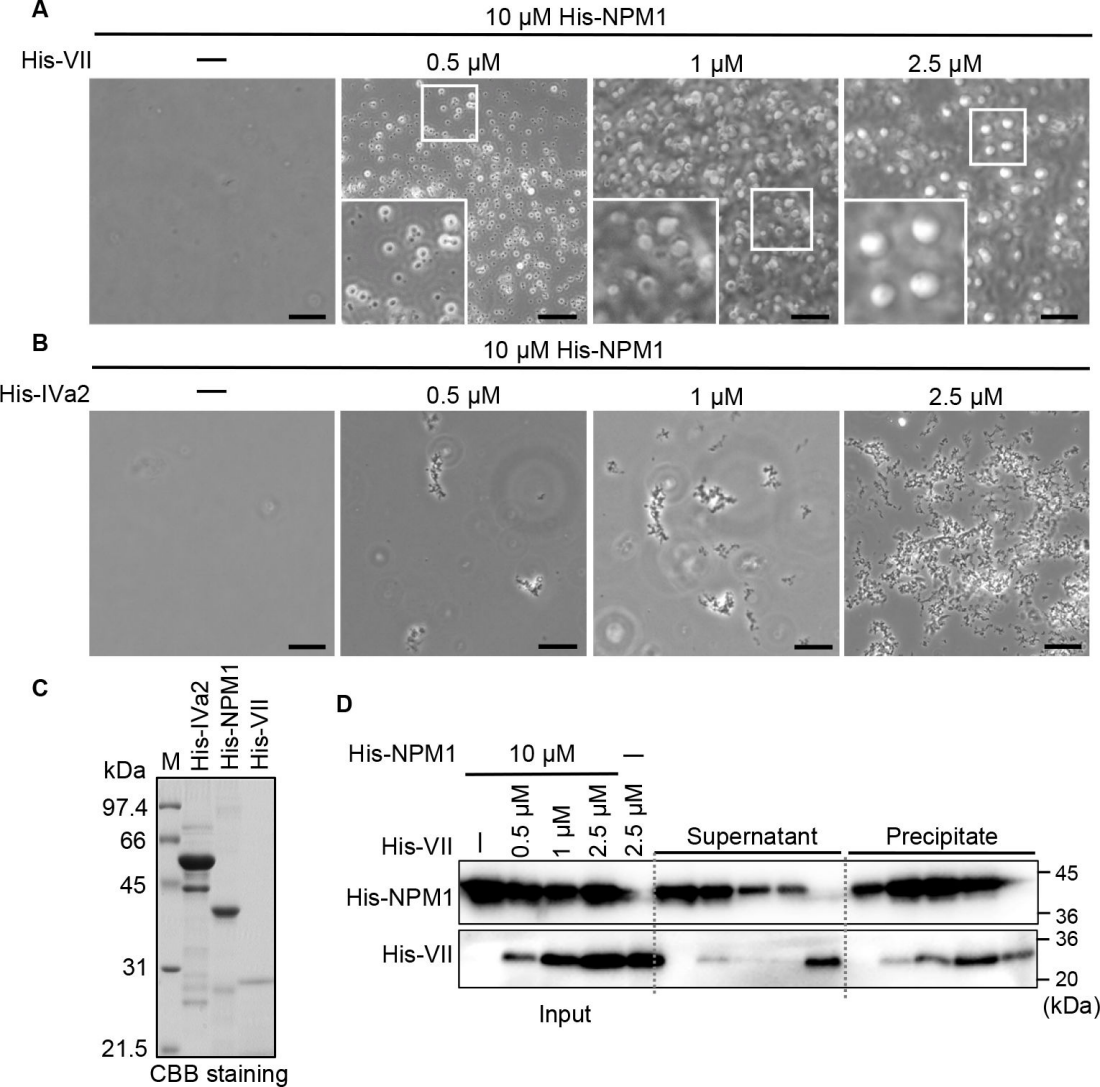
Protein VII triggers heterotypic LLPS of NPM1 *in vitro*. *In vitro* microscopic LLPS assay of 10 µM His-NPM1 mixed with His-protein VII (**A**) and His-protein IVa2 (**B**). The concentrations of Ad proteins were indicated. Enlarged images of liquid droplets formed are shown in the bottom panel. Scale bars, 10 µm. (**C**) Coomassie Brilliant Blue staining of His-IVa2 (2.5 µM), His-NPM1 (10 µM), and His-VII (2.5 µM) used in A and B. (**D**) *In vitro* LLPS sedimentation assay between 10 µM His-NPM1 and 0.5 µM, 1 µM, and 2.5 µM His-VII. The solution was centrifuged to separate the dense (precipitate) and the light (supernatant) phases. The amount of each protein was examined using western blotting analysis with anti-NPM1 and anti-VII antibodies.

NPM1 interacts with viral basic core protein VII as a molecular chaperone for the remodeling of the Ad core (25). NPM1 is also reported to trigger LLPS by interacting with cellular basic proteins such as SURF6 and rpL5 (13, 54). Thus, it is possible that protein VII induces the heterotypic LLPS of NPM1. To address this possibility, we examined the LLPS activity of recombinant NPM1 protein in the presence of either protein VII or IVa2 *in vitro* by microscopic observation. We did not observe the homotypic LLPS of recombinant NPM1 at 10 µM (Fig. 6A). However, the addition of protein VII to NPM1 induced the formation of liquid droplets in a manner dependent on the amount of protein VII. Furthermore, addition of purified Ad DNA to NPM1 and protein VII seem to enhance LLPS evident by increased liquid droplets formed *in vitro* (Fig. S1) suggesting that it is a positive factor in this process. This is in good agreement with previous reports (58, 59) demonstrating that DNA promotes LLPS by acting as a scaffold or binding partner for LLPS-associated proteins through specific interactions between proteins and DNA sequences or through non-specific electrostatic interactions. In contrast, in the presence of protein IVa2, only protein aggregates were observed and no liquid droplets were formed (Fig. 6B). To determine whether the liquid droplets observed in Fig. 6B contain both protein VII and NPM1, we performed an *in vitro* LLPS sedimentation assay by purifying the dense phase and testing it for enrichment of protein VII and NPM1. Figure 6C shows the Coomassie Brilliant Blue staining of the recombinant proteins used in this experiment. The bottom image in Fig. 6D shows turbidity of the solution containing both His-VII and 10 µM His-NPM1. Notably, the amounts of both protein VII and NPM1 were enriched in the precipitate fraction in a protein VII concentration-dependent manner, suggesting that the liquid droplets formed are composed of these proteins.

### NPM1 depletion decreased genome packaging in viral capsids

**Fig 7:**
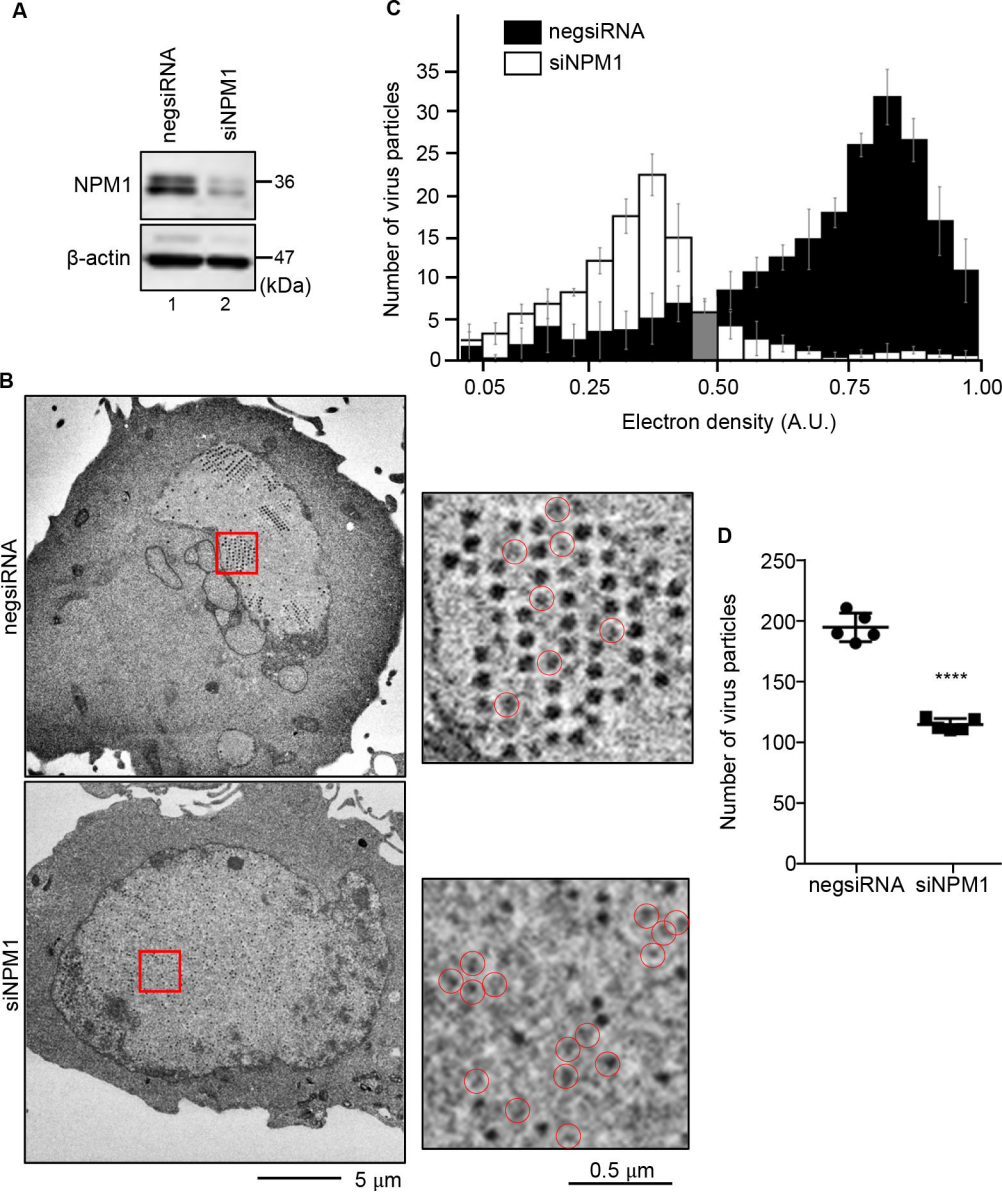
NPM1 depletion decreased the Ad DNA packaging in viral capsids. (**A**) At 36 h post-infection, Ad-infected U2OS cells transfected with either negsiRNA or siNPM1 were absorbed on the copper mesh grids, negatively stained, and then observed by TEM. (**A**) The expressions of NPM1 and B-actin were examined by western blotting. (**B**) Representative images from three independent experiments are shown. Enlarged images of virus particles are shown in the right panel. Red circles indicate the viral capsids with low electron density. Scale bars, 0.5 and 5 µm. (**C**) The distribution of electron density (A.U.) of viral capsids was analyzed and plotted in a histogram. Five nuclei were examined per condition; *n* = 206, 209, 212, 212, and 212 virus particles for negsiRNA and *n* = 121, 111, 119, 105, and 111 virus particles for siNPM1. (**D**) The number of virus particles in negsiRNA- and siNPM1-treated Ad-infected cells was counted and graphed. Error bars indicate ±SD. *P* values were calculated using Student’s *t*-test (****P < 0.0001).

ViPR bodies are reported to cluster together in the periphery of the nucleus around 48 h post-infection, creating another type of membrane-less compartment, the LVAC (41). Transmission electron microscopy (TEM) analysis revealed exclusive paracrystalline viral capsid arrays within the LVAC. We next examined the effect of defective ViPR body formation on virion assembly in NPM1-knockdown U2OS cells by TEM observation. Figure 7A shows that NPM1 was effectively knocked down by NPM1-specific siRNA treatment. Formation of ViPR bodies was significantly decreased by NPM1 depletion at 36 h post-infection (Fig. S2A). Paracrystalline arrays of viral capsids that formed a honeycomb-like pattern were observed at 36 h post-infection in the control cells (Fig. 7B, top panel). In contrast, NPM1 depletion resulted in the disruption of the paracrystalline arrays and dispersed the viral capsids in the nucleoplasm (Fig. 7B, bottom panel). Furthermore, in the control cells, highly electron-dense viral capsids were observed, possibly because the viral genome is packaged into the capsids (Fig. 7C, black bar). However, the level of electron density was reduced to about 50% of that of the control by NPM1 knockdown (Fig. 7C, white bar). Although viral DNA replication and expression levels of several Ad proteins were not affected (Fig. S2B and C), the total number of virus particles significantly decreased in NPM1 knockdown cells (Fig. 7D), which is in good agreement with a previous report that NPM1 knockdown reduces the amount of infectious viral particles (60). Therefore, NPM1-mediated accumulation of Ad core proteins to the ViPR bodies may be important for genome packaging in viral capsids.

## DISCUSSION

**Fig 8:**
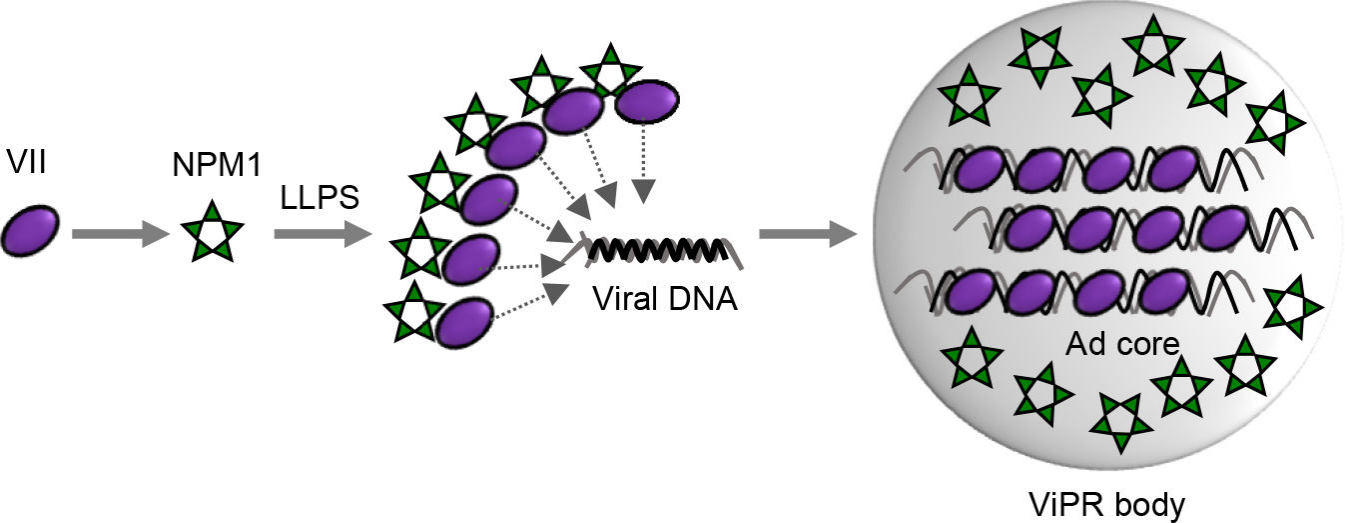
A proposed model. During the late stages of infection, protein VII free of DNA binds to NPM; protein VII induces heterotypic LLPS of NPM1 leading to accumulation of both proteins to the ViPR body; protein VII then dissociates from NPM1 and is deposited to the viral DNA forming the Ad core. Ad core is likely maintained in the ViPR body prior to packaging.

NPM1 reportedly interacts with the core proteins VII and V during the remodeling of the Ad core. In this process, similarly to its function as a histone chaperone, NPM1 regulates the association/dissociation of Ad core proteins and cellular histones with the replicated virus genome for proper Ad core assembly. In this study, we found that NPM1 and protein VII accumulate in the ViPR bodies through LLPS (Fig. 4) and that the interaction between these proteins triggers LLPS *in vitro* (Fig. 6). Other cellular chromatin regulatory proteins, including NCL and UBF, reportedly also accumulate in the ViPR bodies (22). Therefore, after the LLPS-mediated deposition of NPM1 and protein VII to the ViPR bodies, this compartment may serve as a nuclear domain for the remodeling of Ad chromatin (Fig. 8).

Recently, other groups have attempted to purify Ad replication compartments (52) and found enrichment of host factors involved in ribosome biogenesis, stress response, and DNA repair. Similarly, we observed enrichment of nucleolar ribosome biogenesis-related factors such as Mybbp1a, NPM1, NCL, and FBL in the fraction containing the ViPR bodies. However, host factors such as MCM2 and TOPOI were not detected in the fractions, suggesting that replication compartments were not co-fractionated with the ViPR body. Nevertheless, the differences in host factor enrichment observed in the purification of Ad replication compartments and ViPR body highlight the importance of examining the proteome to identify cellular processes manipulated during viral replication.

Intriguingly, the localization patterns of the nucleolar proteins NPM1 and FBL within the ViPR body is reversed of their distribution in the nucleolus. Fibrillarin is primarily localized in the dense fibrillar component (DFC) of the nucleolus where it plays a crucial role in ribosomal RNA (rRNA) processing, specifically in the methylation of rRNA (61, 62). On the other hand, NPM1 has a broader localization pattern within the nucleolus as it is found in the nucleoplasm, granular component (GC), and DFC of the nucleolus, where it is involved in multiple processes, including ribosome biogenesis, RNA processing, and nucleolar formation (63, 64). The reversal of NPM1 and FBL’s localization in the nucleolus and ViPR body could be correlated to their LLPS activity. NPM1 has been shown to undergo LLPS with ribosomal RNA (rRNA) and other nucleolar proteins, forming membrane-less condensates within the nucleolar environment. These condensates may serve as sites for the concentration and assembly of ribosomal subunits and other factors involved in ribosome biogenesis (13). On the other hand, FBL’s LLPS activity could contribute to the formation of higher-order assemblies involved in pre-rRNA processing (8). These assemblies might serve as sites for efficient modification and processing of rRNA transcripts, ultimately contributing to ribosome assembly and maturation. This difference underscores the dynamic nature of subcellular compartmentalization and the potential functional implications of protein localization shifts between cellular structures like the nucleolus and the ViPR body. It is possible that while NPM1’s LLPS activity enables the formation of condensates to concentrate and assembly factors in the ViPR body, FBL may support NPM1’s LLPS activity by forming an outer shell to scaffold the ViPR body.

NPM1 has four major functional domains: an N-terminal core/oligomerization domain (1–120 a.a.), an acidic region (121–188 a.a.), a basic region (189–243 a.a.), and a folded globular domain (254–294 a.a.) (65). NPM1 forms pentamers via its oligomerization domain, and these pentamers engage in hydrophobic interactions forming decamers and higher-order oligomers (66). The pentameric NPM1 binds to arginine/lysine-rich positively charged motifs of other proteins via its acidic region, triggering heterotypic LLPS (54). Protein VII is the major protein component of the Ad core and contains a highly arginine-rich region (RRR motif) of about 20 amino acid residues in the middle region (55). Although the details of the binding mechanism between NPM1 and protein VII remain unclear, the acidic tracts of pentameric NPM1 likely interact with the RRR motif of protein VII through electrostatic interactions, triggering heterotypic LLPS. NPM1 has also been reported to bind to protein VII free of DNA in Ad-infected cells as a molecular chaperone (40). The RRR motif recognizes the phosphate DNA backbone, and the amino and carboxyl termini of protein VII bind to DNA in cooperation with the RRR motif (67, 68). These findings suggest that the DNA-binding region and the putative region for LLPS formation within protein VII partially overlap. Thus, the hypothetical model of protein VII and NPM1 interaction in the ViPR bodies is as follows: protein VII free of DNA binds to NPM1 through the RRR motif; protein VII induces heterotypic LLPS of NPM1 leading to accumulation of both proteins to the ViPR body; protein VII then dissociates from NPM1 and is deposited to the viral DNA (Fig. 8).

In this study, although 1,6-hexanediol treatment disrupted the accumulation of both NPM1 and protein VII in the ViPR body, DNA accumulation was still observed. It has been revealed that 1,6-HD treatment induces excessive condensation of chromatin in human cells, as observed through live-cell single-nucleosome imaging (69). Additionally, *in vitro* experiments demonstrated that 1,6-HD notably facilitates cation-dependent chromatin condensation, distinct from its ability to melt liquid droplets. Major core protein VII assembles Ad DNA into a nucleosome-like structure called the Ad core. Thus, it is possible that 1,6-HD treatment induced compaction of Ad core, which could partly explain why the accumulated DNA in the ViPR body was undisrupted.

Knockdown of NPM1 reduced the electron density of the viral capsids and dispersed the capsids into the nucleoplasm (Fig. 7). Although the details of this mechanism remain unknown, it is proposed that the viral capsids are assembled in close proximity to the Ad cores in LVAC and that these capsids are then stably deposited as paracrystalline viral capsid arrays (41). This suggests that the accumulation of viral core proteins within ViPR bodies may serve as a scaffold for viral capsid assembly, facilitating the encapsidation of viral DNA. Furthermore, it has been reported that depletion of NPM1 causes the viral DNA to interact with an excess amount of core proteins (40). It is also possible that the defective Ad cores are not packaged into the viral particles through an unknown quality control mechanism of the Ad core. To further elucidate the mechanism of NPM1-mediated viral particle formation, understanding the packaging mechanism of the Ad core into viral capsids is required.

## MATERIALS AND METHODS

### Biological materials

U2OS, EGFP-NPM1 HeLa, and EGFP-Histone H3 HeLa cells were maintained in Dulbecco’s modified Eagle’s medium (DMEM; Nissui Pharmaceutical) containing heat-inactivated 10% fetal bovine serum (FBS). The human adenovirus type 5 (HAdV5) used in this study was amplified and purified as described previously (70). Cells to be infected were plated in culture dishes 1 day prior to infection and maintained in DMEM containing 10% FBS. Cells were infected with HAdV5 in DMEM without FBS for 1 h at a multiplicity of infection (MOI) of 10–20. After washing with DMEM without FBS, cells were cultured at 37°C in DMEM containing 5% FBS.

### Antibodies

Rabbit polyclonal antibodies against NPM1, protein VII, and IVa2 were prepared as previously described (71–73). A mouse monoclonal antibody against DBP was generously provided by Dr W. C. Russell (University of St. Andrews, Fife, UK). Mouse monoclonal anti-FBL (H-140), anti-β-actin (C4), anti-TOPOI (H5), anti-RPA194 (C1), and anti-UBF (H-300) were purchased from Santa Cruz Biotechnology. Goat anti-MCM2 antibody (N-19) was obtained from Santa Cruz Biotechnology. Anti-H2A and anti-Mybbp1a (14524–1-AP) antibodies were obtained from Abcam and Proteintech, respectively. Mouse RNA pol II (p-Ser2) MABI0602 was purchased from Cosmo Bio, USA.

### Preparation of subnuclear fractions enriched with ViPR bodies

U2OS cells were infected with HAdV5 at an MOI of 20. At 24 and 36 h post-infection, the Ad-infected U2OS cells were harvested and resuspended in ice-cold hypotonic buffer [10 mM HEPES (pH 7.9), 10 mM KCl, 1.5 mM MgCl_2_, 0.5 mM DTT, 20 µg/mL phenylmethylsulfonyl fluoride (PMSF), 10 µg/mL bovine pancreatic trypsin inhibitor, 10 µg/mL pepstatin A, and 10 µg/mL leupeptin]. The cells were homogenized with a loose-fitting A-type pestle homogenizer. The cell homogenates were centrifuged at 300 × *g*, 4°C for 5 min to isolate the nuclei as a pellet. The isolated nuclei were resuspended in solution 1 (S1; 0.25 M sucrose, 10 mM MgCl_2_ 20 µg/mL PMSF, 10 µg/mL bovine pancreatic trypsin inhibitor, 10 µg/mL pepstatin A, and 10 µg/mL leupeptin) and layered over with an equal volume of solution 2 (S2; 0.35 M sucrose, 0.5 mM MgCl_2_, 20 µg/mL PMSF, 10 µg/mL bovine pancreatic trypsin inhibitor, 10 µg/mL pepstatin A, and 10 µg/mL leupeptin). After centrifugation at 1,400 × *g*, 4°C for 5 min, the isolated nuclei were collected as a pellet. The isolated nuclei were resuspended in S2 and sonicated in a cold ultrasonic bath. The sonicated sample was layered over with an equal volume of solution 3 (S3; 0.88 M sucrose, 0.5 mM MgCl_2_) and centrifuged at 3,000 × *g*, 4°C for 10 min. The supernatants were again layered with an equal volume of solution 3 (S3; 0.88 M sucrose, 0.5 mM MgCl_2_) and centrifuged at 3,000 × *g*, 4°C for 10 min. The supernatants and pellets were collected as the nucleoplasm and the ViPR body fractions, respectively. The amount of Ad DNA in the subnuclear fractions was examined by qPCR with primers specific for the E1A promoter region (5′-GGGTCAAAGTTGGCGTTTTA-3′ and 5′-CAAAATGGCTAGGAGGTGGA-3′). The isolated fractions were subjected to western blot analysis with indicated antibodies. liquid chromatography-mass spectrometry (LC-MS) analysis was performed using a Q Exactive HF-X mass spectrometer (Thermo Fisher Scientific) four times for each sample and two biological replicates and two technical replicates per sample. The peptides were identified using SEQUEST HT, and the abundance of each peptide was calculated by ProteinDiscoverer 2.2 software. The nuclear proteins that were consistently detected in each of the measurements were applied to Gene Ontology annotations using the DAVID database. The relative abundance was calculated by dividing the abundance of each peptide obtained at 24 or 36 hpi by that of uninfected sample. The *P* value was determined by an unpaired two-tailed Student’s *t*-test.

### Indirect immunofluorescence assays

Indirect immunofluorescence assays were carried out according to a previously described protocol (74). Briefly, Ad-infected cells were fixed in 1.5% paraformaldehyde in PBS for 10 min at room temperature. The fixed cells were then permeabilized with PBS containing 0.5% Triton X-100 for 20 min at room temperature. After incubation in 1% non-fat milk in PBS containing 0.1% Triton X-100 for 15 min, the coverslips were incubated with primary antibodies for 1 h, which was followed by incubation with secondary antibodies (AlexaFluor 488-conjugated anti-rabbit IgG and AlexaFluor 568-conjugated anti-mouse IgG; Thermo Fisher Scientific) for 1 h in the dark. The intracellular localization of the target proteins was observed by use of confocal laser-scanning microscopy (LSM700; Carl Zeiss). The fluorescence intensity line profiles were examined by NIH images. The values of the fluorescence intensities were represented as means ± standard deviations.

### Fluorescence recovery after photobleaching analysis

HeLa cells stably expressing EGFP-NPM1 or EGFP-H3 were infected with HAdV5 at an MOI of 20. At 36 h post-infection, the ViPR body was bleached using 100% transmission of a 488-nm laser, and the images (512 × 512 pixels; scan speed 8) were collected every 300 ms for 120 sec. The fluorescence recovery data were normalized using data acquired from the non-bleached regions. To calculate the half-recovery time, the FRAP curves were fitted with the one-phase exponential equation using GraphPad Prism (version 10.2.3).

### *In vitro* microscopic LLPS assay

His-NPM1, His-protein VII, and His-protein IVa2 were cloned into pET14b (71, 73) and expressed in the *Escherichia coli* strain BL21(DE3). The recombinant proteins were purified according to the manufacturer’s protocol. For His-protein VII, the protein was solubilized with 6 M urea before Ni-NTA purification.

His-protein VII was solubilized with 6 M urea before Ni-NTA purification. The purified protein was then dialyzed against water at 4°C overnight. The precipitate formed was collected and dissolved in a buffer containing 20 mM Tris-HCl (pH 7.9), 0.5 M NaCl, and 6 M urea and further purified through HiPrep Sephacryl S-200 column chromatography (Amersham Biosciences). The purified His-VII was dialyzed against water and stored at −30°C until use.

The purified His-NPM1 and His-protein IVa2 were dialyzed against a buffer containing 50 mM HEPES-NaOH (pH 7.5), 200 mM NaCl, 1 mM EDTA, and 1 mM DTT. Microscopic examination of the *in vitro* LLPS activity was performed by loading of the indicated concentrations of recombinant His-NPM1, His-VII, or His-IVa2 onto a 35-mm-diameter glass-bottom dish. After incubation for 5 min, the solution was examined under a phase contrast microscope for liquid droplet formation.

### *In vitro* sedimentation LLPS assay

Indicated concentrations of recombinant His-NPM1 and His-VII were mixed in a 1.5-mL test tube. After incubation for 5 min, the solution was centrifuged for 5 min at 13,000 rpm/min. The precipitate (dense phase) was resuspended in a volume of LLPS buffer equal to the supernatant (light phase). The precipitate and supernatant fractions were then loaded onto SDS-PAGE for western blotting analysis with the appropriate antibodies.

### Immunoprecipitation analysis of NPM1 and Ad VII interaction

At 36 h post-infection, Ad-infected U2OS cells were subjected to either 3.8% 1,6-HD or 2,5-HD treatment for 90 sec. The cells were then collected and resuspended in an immunoprecipitation buffer [50 mM HEPES-NaOH (pH 7.9), 200 mM NaCl, 0.1% Triton X-100] and lysed by sonication. After centrifugation at 13,000 × *g*, 4°C for 10 min, the lysates were mixed with anti-IgG or anti-NPM1 antibody and incubated at 4°C overnight. Protein A-Sepharose beads (GE Healthcare) were added, and the mixture was further incubated at 4°C for 2 h. The beads were washed three times with immunoprecipitation buffer. SDS sample buffer was added to elute the immunoprecipitated proteins.

### Gene silencing mediated by siRNA

Short interfering RNA (siRNA) against the *NPM1* gene (HSS143154; Thermo Fisher Scientific) and negative control siRNA were purchased. U2OS cells were transfected with siRNA by use of Lipofectamine RNAi MAX (Thermo Fisher Scientific) according to the manufacturer’s protocol.

### Transmission electron microscopy

Ad-infected cells were fixed with 2.5% glutaraldehyde at 4°C overnight. After further fixation with 1% OsO_4_ for 30 min at 4°C, sequential dehydrations with ethanol in a stepwise manner were carried out, which was followed by propylene oxide treatment, and the samples were then embedded in Epon. Ultrathin slices were examined with a JEM-1400 transmission electron microscope (JEOL).

### Statistical analysis

Values were represented as the means ± SDs of three independent experiments. The significance of the experimental results was determined by an unpaired two-tailed Student’s *t*-test using GraphPad Prism (version 7.03). ****P < 0.0001.

## SUPPLEMENTAL MATERIAL

Supplemental figures spectrum.00416-24-s0001.pdf Fig. S1 and S2.

Table S1 spectrum.00416-24-s0002.xlsx Proteomics data of ViPR body fraction at 0, 24, and 36 h-post infection analyzed by LC-MS.

ASM does not own the copyrights to Supplemental Material that may be linked to, or accessed through, an article. The authors have granted ASM a non-exclusive, world-wide license to publish the Supplemental Material files. Please contact the corresponding author directly for reuse.
