# Supplementary material for: Nuclear reorganization by NPM1-mediated phase separation triggered by adenovirus core protein VII
Source: Microbiol Spectr. 2024 Aug 20;12(10):e00416-24. doi: 10.1128/spectrum.00416-24 (PMC11448090; doi:10.1128/spectrum.00416-24)
Supplement: Supplemental figures — Fig. S1 and S2. [file spectrum.00416-24-s0001.pdf]

Nuclear reorganization by NPM1-mediated phase separation triggered by adenovirus core protein VII

Michelle Jane Genoveso[a], Mitsuru Okuwaki[b], Kohsuke Kato[a], Kyosuke Nagata[a], Atsushi Kawaguchi[a,c,d,e]#

[a] Department of Infection Biology, Institute of Medicine, University of Tsukuba, Japan

[b] Laboratory of Biochemistry, School of Pharmacy, Kitasato University, Tokyo, Japan

[c] Transborder Medical Research Center, University of Tsukuba, Japan

[d] Microbiology Research Center for Sustainability, University of Tsukuba, Tsukuba, Japan

[e]Center for Quantum and Information Life Sciences, University of Tsukuba, Tsukuba, Japan

#Address correspondence to Atsushi Kawaguchi, ats-kawaguchi@md.tsukuba.ac.jp

Supplementary Figure 1

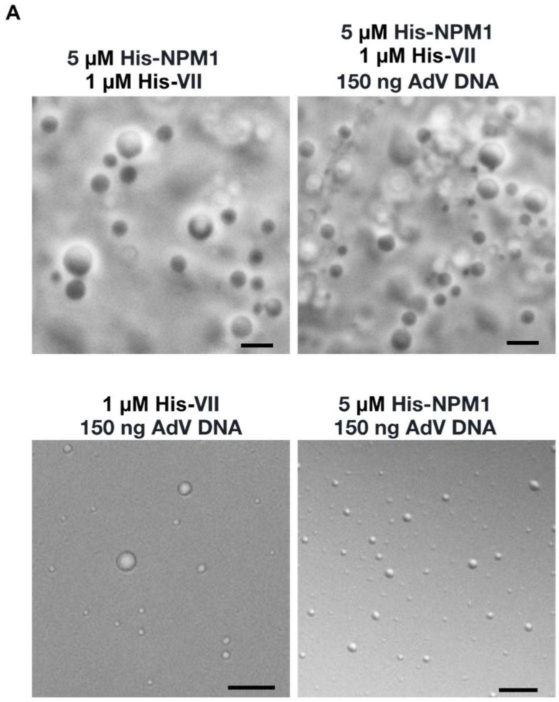

SUPP FIG 1. Ad DNA enhances heterotypic LLPS of NPM1 and protein VII *in vitro.*

In vitro microscopic LLPS assay of His-NPM1, His-protein VII and purified Ad viral DNA. The concentrations of proteins and Ad DNA were indicated. Scale bars, 10 μm.

Supplementary Figure 2

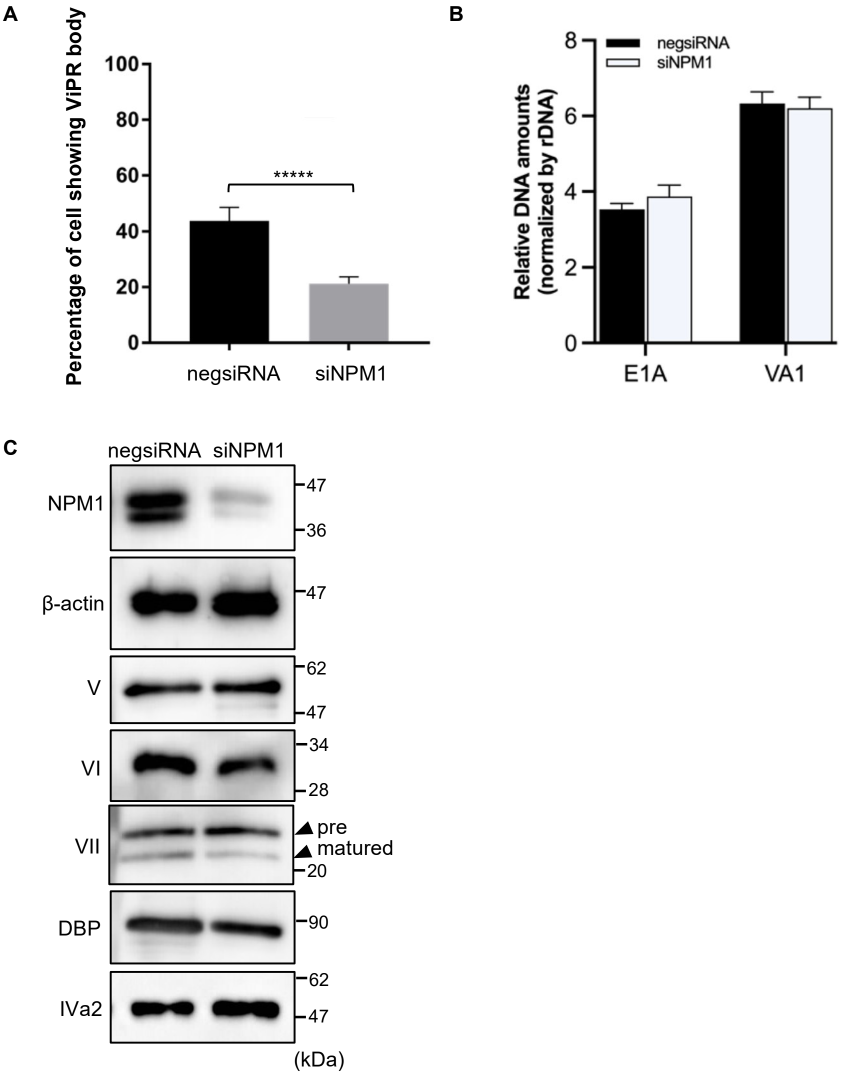

SUPP FIG 2. NPM1 depletion delayed the formation of ViPR bodies but did not affect viral DNA replication and protein expression.

(A) Control and NPM1-depleted cells were infected with HAdV5, and at 36 h posi-infection, cells were subjected to IF analysis with anti-DBP and anti-NPM1 antibodies and co-stained with DAPI to visualize DNA. Under our assay condition, most cells do not show ViPR bodies when treated with NPM1 siRNA. Cells showing ViPR body were counted and their population was graphed in percentage. Experiments were performed five times and the number of cells counted were as follows: for control siRNA-treated cells, n = 123, 135, 118, 136, and 114; for NPM1 siRNA-treated cells, n = 156, 125, 138, 155, and 129. Error bars indicate +/- SD. P-values were calculated using student's t-test (*****P < 0.00001). (B) Quantitative analyses of Ad genomic DNA. U2OS cells were mock- or

HAdV5-infected with MOI of 20. At 36 hpi, DNA was purified from the cells and the amounts of viral DNA were examined by qPCR using a primer set specific for the E1A and VA1 regions. The relative amounts of Ad DNA were normalized to that of rDNA and shown in graph. Filled and blank bars indicate the DNA amounts from control cells and NPM1 siRNA-treated cells, respectively. (C) Control and NPM1 siRNA-treated U2OS cells (lanes 1 and 2, respectively) were infected with HAdV5 (MOI = 20) at 60 h post siRNA transfection. At 36 hpi, cells were collected and the expression of Ad proteins, NPM1 and β-actin was examined by western blotting. Positions of molecular weight markers are shown at the right side of the panels.
